# Enhancing glaucoma prediction across ancestries: integrating functional annotation into multi-trait polygenic risk scores

**DOI:** 10.3389/fgene.2026.1842316

**Published:** 2026-05-07

**Authors:** Xiaoyi Raymond Gao

**Affiliations:** 1 Department of Ophthalmology and Visual Sciences, The Ohio State University, Columbus, OH, United States; 2 Department of Biomedical Informatics, The Ohio State University, Columbus, OH, United States

**Keywords:** diverse ancestry, genetic prediction, glaucoma, polygenic risk score, risk stratification

## Abstract

**Introduction:**

Primary open-angle glaucoma (POAG) remains a leading cause of irreversible blindness worldwide, with early detection crucial for preventing vision loss. Current polygenic risk scores (PRS) for POAG, however, demonstrate limited predictive power.

**Methods:**

Here, we present a multi-trait polygenic probability risk score (PPRS) approach that integrates PRSs of multiple glaucoma-related traits, including POAG, intraocular pressure (IOP), vertical cup-to-disc ratio (VCDR), and retinal nerve fiber layer thickness, while leveraging functional genomic annotations and extensive genomic coverage (>7 million variants) to enhance POAG prediction across diverse ancestries. We evaluated the PPRS in the UK Biobank (n = 324,713, European ancestry) and the Mexican American Glaucoma Genetic Study (MAGGS, n = 4,549, Latino ancestry).

**Results:**

The PPRS improved prediction compared with conventional approaches, achieving area under the receiver operating characteristic curve (AUC) values of 0.814 in Europeans and 0.802 in Latinos, versus 0.721 and 0.753 for baseline models with age and sex alone. Genetic contributions varied by ancestry: IOP PRS showed the strongest association in Europeans (OR = 1.63, P = 5.37 × 10^−89^), whereas VCDR PRS predominated in Latinos (OR = 1.64, P = 2.04 × 10^−11^). Risk stratification was substantial, with the highest PPRS decile showing 74.4-fold and 49.3-fold greater POAG risk compared with the lowest decile in Europeans and Latinos, respectively.

**Discussion:**

These findings show that integrating multiple disease-relevant PRSs and functional annotations significantly improves genetic prediction of POAG across diverse populations, supporting applications in targeted screening and early intervention.

## Introduction

Glaucoma is the leading cause of irreversible blindness (Fan and Wiggs, 2010; Blindness et al., 2021), affecting about 80 million individuals worldwide (Weinreb et al., 2016; Tham et al., 2014), with primary open-angle glaucoma (POAG) constituting approximately 90% of glaucoma cases in North America (Tham et al., 2014; Lang, 2007). Despite available treatments to slow disease progression, the irreversible nature of glaucoma-induced vision loss underscores the critical importance of early detection and intervention. While traditional risk assessment relies primarily on clinical measurements and family history, recent advances in genomics offer promising opportunities for enhanced risk stratification.

Polygenic risk scores (PRSs) have emerged as powerful tools for quantifying genetic predisposition to complex diseases (Purcell et al., 2009; Kachuri et al., 2024; Torkamani et al., 2018). However, their application to POAG has faced several key limitations. First, previous studies utilizing single-trait PRSs have achieved limited stratification ability, with only about 50% of cases being identified in the top PRS quintile in a recent report (de Vries et al., 2025). Second, existing approaches have limited capacity to capture the inherent complexity of POAG’s pathophysiology, which involves multiple quantitative endophenotypes (Fan and Wiggs, 2010; Ramdas et al., 2011), including intraocular pressure (IOP), vertical cup-to-disc ratio (VCDR), and retinal nerve fiber layer (RNFL) thickness. The multi-trait analysis of genome-wide association studies (MTAG) approach can increase power to detect associations by jointly analyzing genetically correlated traits; however, it assumes that all SNPs share the same variance-covariance matrix across traits, potentially leading to biased effect size estimates and an increased rate of false positives (Turley et al., 2018). Third, most POAG PRS prediction studies have employed limited genomic coverage (<1M markers) and have lacked integration of functional annotations. These limitations highlight the necessity for more sophisticated models that can capture the multifaceted genetic underpinnings of POAG.

Here, we present a multi-trait polygenic probability risk score (PPRS) approach that addresses these limitations through three key innovations: 1) integrating multiple disease-relevant quantitative traits into PRS modeling while preserving the individual contributions of each trait to enable interpretable, ancestry-specific risk assessment; 2) incorporating extensive genomic coverage (>7 million variants) and functional annotations; 3) validating across diverse ancestral populations. We hypothesized that this comprehensive approach would significantly improve POAG risk stratification across diverse populations compared to existing methods. To test this hypothesis, we evaluated our model in two independent cohorts: the UK Biobank (UKB) for European ancestry individuals and the Mexican American Glaucoma Genetic Study (MAGGS) for Latino participants.

## Materials and methods

### Study design and populations

We conducted a two-stage study: first, deriving genome-wide association study (GWAS) summary statistics from previously published data and our own GWAS analyses (details provided below); second, constructing (incorporating functional annotations) and evaluating PRSs in a subset of the UKB cohort (n = 324,713, POAG cases: 2,168) and the MAGGS cohort (n = 4,549, POAG cases: 275), both independent of the GWAS discovery samples. UKB was approved by the North West Multi-Center Research Ethics Committee (Sudlow et al., 2015; Allen et al., 2014) and MAGGS was approved by the institutional review board at the University of Illinois at Chicago (Nannini et al., 2017). All participants provided written informed consent. The study adhered to the tenets of the Declaration of Helsinki. We obtained fully de-identified data to ensure participant confidentiality. We utilized the UKB and MAGGS datasets because both include genome-wide genotyping and detailed ophthalmic phenotypes suitable for glaucoma risk modeling. The UKB provided a large cohort enabling development and benchmarking of predictive models in participants of European ancestry, while MAGGS offered an independent Latino cohort for cross-ancestry evaluation.

### UKB dataset

The UKB is an ongoing, large-scale prospective cohort study with over 500,000 adult participants aged 40 to 69 at enrollment (2006–2010), registered with the National Health Service in the United Kingdom (Sudlow et al., 2015; Allen et al., 2014). The study collected medical information and DNA samples, including ophthalmologic data for a subset of approximately 118,000 participants. Our analysis focused on participants of European ancestry. Genotyping was performed using the UK BiLEVE Axiom Array or the UKB Axiom Array, with imputation based on reference panels from the 1000 Genomes Project (1KGP), UK10K, and the Haplotype Reference Consortium (Bycroft et al., 2018). Variants with an imputation INFO score less than 0.3 and minor allele frequency less than 0.01 were excluded, resulting in approximately 10.3 million variants for downstream analysis. For individuals with IOP measurements, VCDR values, and RNFL data, we used previously published IOP GWAS results (Gao et al., 2018), VCDR summary statistics (Han et al., 2021), and conducted a GWAS of RNFL to derive summary statistics. For those without ophthalmic exams (n = 324,713, unrelated, independent of the IOP, VCDR, and RNFL GWAS samples) (Gao et al., 2019), we utilized their data for PRS construction and association testing with POAG. Importantly, individuals contributing to the IOP, VCDR, and RNFL GWAS were distinct from those used as the target sample for PRS testing, ensuring no sample overlap. POAG (H40.1) cases were identified using the International Classification of Diseases Tenth Revision (ICD-10) codes. Controls were identified as those who did not have glaucoma. Because the UKB relied on ICD-10 diagnostic codes recorded during routine clinical encounters rather than standardized ophthalmic screening, case ascertainment depends on participants having accessed eye care. The UKB cohort was drawn from the National Health Service, which provides universal healthcare coverage; however, the extent of glaucoma-specific screening varied among participants. Moreover, glaucoma cases were on average older than controls (63.0 vs. 57.1 years), consistent with the age-dependent nature of POAG. We did not perform age-matching because age is a key predictor included as a covariate in all regression models, and matching on age would have reduced statistical power while limiting the generalizability of our findings to the broader population distribution represented in the UKB.

### MAGGS dataset

MAGGS samples were sourced from the Los Angeles Latino Eye Study (LALES) (Varma et al., 2004a), a population-based epidemiologic study examining visual impairment and ocular diseases in 6,357 Latino individuals aged 40 or older in Los Angeles County, California. The presence of POAG was determined by agreement among three glaucoma specialists using ophthalmologic examination data, including the presence of open angles, characteristic visual field abnormalities, and optic disc damage in at least one eye. MAGGS and LALES genotyped 4,996 Latino individuals using either the Illumina OmniExpress BeadChip or the Illumina Hispanic/SOL BeadChip (Nannini et al., 2017; Nannini et al., 2016). Imputation was performed using 1KGP reference panel, excluding variants with a Mach (Li and Abecasis, 2006) Rsq less than 0.3 and minor allele frequency less than 0.01, resulting in approximately 9.9 million imputed variants. Our analysis included 4,549 participants (4,108 unrelated and 441 related individuals) from this dataset. Importantly, LALES was a population-based study in which all participants underwent standardized comprehensive ophthalmic examinations, including visual field testing and optic disc assessment, regardless of prior diagnosis or insurance status. Consequently, POAG case ascertainment in MAGGS was based on clinical examination by glaucoma specialists rather than self-report or insurance-dependent diagnostic codes. Cases were on average older than controls (65.9 vs. 56.2 years), reflecting the age-dependent prevalence of POAG. Age was included as a covariate in all analyses rather than employing age-matching, for the same reasons described above for the UKB cohort.

### GWAS summary statistics

We used previously published GWAS summary statistics for POAG, IOP, and vertical cup-to-disc ratio (VCDR), as well as our own GWAS results for RNFL. We primarily selected publicly available GWAS summary statistics with the largest available sample sizes at the time of analysis. When suitable public datasets were unavailable, we conducted in-house GWAS to generate summary statistics. This strategy ensured the use of the most comprehensive and up-to-date data for each phenotype. The details are as follows.

1. POAG: We used GWAS results from Gharahkhani et al. (2021), which performed a multi-ethnic meta-analysis using the International Glaucoma Genetics Consortium (IGGC) and UKB datasets, comprising 383,500 individuals of European, Asian, and African descent (Gharahkhani et al., 2021). For building PRSs for UKB participants, we used results from IGGC only, excluding UKB individuals, resulting in a cross-ancestry meta-analysis with 192,702 individuals suitable for UKB PRS construction.
2. IOP: We used GWAS results from Gao et al. (2018), including 115,486 UKB participants of European ancestry (Gao et al., 2018).
3. VCDR: We used GWAS summary statistics from Han et al. (2021), involving 111,724 individuals of European and Asian descent from UKB, the Canadian Longitudinal Study on Aging (CLSA), and IGGC (Han et al., 2021). VCDR measurements were obtained from human graders and deep learning models applied to retinal fundus images.
4. RNFL: We conducted a GWAS with 52,902 European ancestry UKB participants using the REGENIE (Mbatchou et al., 2021) software and RNFL thickness values derived from optical coherence tomography images (Ko et al., 2017). We excluded outliers and low-quality images (image quality score less than 45). We averaged RNFL values between the left and right eyes and applied rank-based inverse normalization. We adjusted for age, sex, diabetes, spherical equivalent, and the first 10 principal components of genetic ancestry, as these factors are known to influence RNFL thickness or serve as potential confounders in genetic association studies. Supplementary Figure 1 presents a Manhattan plot of the genome-wide -log10(*P* values) for our RNFL GWAS analysis.

These summary statistics provided the foundation for constructing PRSs in our analysis, tailored to different phenotypes relevant to POAG risk.

### PRS construction

We employed three different approaches to construct PRSs:

First, we implemented a traditional clumping and thresholding (C + T) based PRS (Prive et al., 2019) using PLINK (Purcell et al., 2007; Chang et al., 2015) to construct the POAG PRS, denoted as PRS-POAG-C + T. Independent single nucleotide polymorphisms (SNPs) were identified using linkage disequilibrium (LD)-based clumping (*r* ^2^ < 0.3) and selected based on a p-value threshold of *P* < 5 × 10^−5^.

Second, we constructed enhanced PRSs using the SBayesRC (Zheng et al., 2024) method, which leverages functional genomic annotations and extensive SNP coverage to improve polygenic risk prediction for complex traits or diseases. By integrating 96 functional annotations from BaselineLD (v2.2) (Zheng et al., 2024; Gazal et al., 2017), including regulatory elements, chromatin states, and transcription factor binding sites, and analyzing over seven million SNPs, we sought to substantially improve PRS accuracy. SBayesRC was implemented using the UKB EUR imputed LD reference panel with default model parameters. We constructed PRSs for POAG (PRS-POAG), IOP (PRS-IOP), VCDR (PRS-VCDR), and RNFL thickness (PRS-RNFL).

Third, we constructed a MTAG-derived PRS for POAG to enable direct comparison. We utilized summary statistics for 2,763 uncorrelated SNPs (identified through LD clumping at *r* ^2^ = 0.1 and a significance threshold of *P* < 0.001) from Craig *et al.*'s (2020) MTAG analysis (Craig et al., 2020). The MTAG-derived PRS was calculated using the formula: PRS-POAG-MTAG(i) = ∑ β_j_ G_ij_, where β_j_ represents the regression coefficient for SNP_j_, and G_ij_ indicates the risk allele dosage for SNP_j_ in individual i. Since the MTAG summary statistics were generated using UKB data, we avoided applying the MTAG-derived PRS to UKB participants to prevent data leakage. Consequently, this PRS was exclusively applied to MAGGS participants.

### Statistical analysis

For testing the association between the PRSs and POAG, we carried out traditional logistic regression analyses, adjusting for age and sex. All constructed PRSs were standardized to have a mean of zero and a variance of one. To check potential multicollinearity among PRSs, we used two complementary approaches. First, we computed the variance inflation factor (VIF) for each predictor, noting that VIF values less than 5 typically indicate low correlation (James et al., 2009). Second, we employed XGBoost (Chen and Guestrin, 2016), a tree-based method known to be less sensitive to collinearity, and examined feature importances via SHapley Additive exPlanations (SHAP) values (Lundberg et al., 2020), following our previous methodology (Gao et al., 2023).

For assessing the predictive ability of the PRSs for POAG, we used penalized (L2) logistic regression with stratified 10-fold cross-validation performed separately in the UKB and MAGGS datasets. We defined the polygenic probability risk score (PPRS) as the predicted probability output from an L2-penalized logistic regression model (scikit-learn LogisticRegression with penalty = “l2” (Pedregosa et al., 2011)), which integrates four PRSs (PRS-POAG, PRS-IOP, PRS-VCDR, and PRS-RNFL) together with age and sex as covariates. The model takes these inputs and outputs a probability of glaucoma for each individual, which we term the PPRS. This formulation differentiates the PPRS from traditional single-trait PRSs and the MTAG-derived PRS. For MAGGS related samples, predictive models were developed using MAGGS unrelated samples and subsequently applied to the related individuals to derive predicted probabilities. We used the area under the receiver operating characteristic curve (AUC) to quantify the predictive ability of the PPRS on POAG. Pairwise comparisons of AUC between different models were performed using Delong’s test (DeLong et al., 1988). For risk stratification, we divided the PPRS into deciles and compared the odds of POAG among study participants in higher PPRS deciles *versus* those in the lowest PPRS decile in both the UKB and MAGGS datasets. A statistical significance threshold of *P* < 0.05 was used for all analyses. All analyses were performed using R (v4.4; including the ggplot2 package for plotting), SAS 9.4 (SAS, Inc., Cary, NC, for non-penalized logistic regression), and Python (v3.12; scikit-learn for penalized logistic regression, XGBoost for gradient boosting models, and SHAP for model interpretability).

## Results

Table 1 summarizes the characteristics of our study samples from UKB and MAGGS. The UKB cohort comprised 324,713 individuals with 2,168 glaucoma cases and 322,545 controls. The mean (standard deviation [SD]) age of glaucoma and non-glaucoma was 63.0 (5.7) and 57.1 (8.0) years, respectively. Females consisted of 48.4% of glaucoma cases and 54.3% of controls. The MAGGS dataset included 4,549 participants with 275 glaucoma cases and 4,274 controls. The mean (SD) age of glaucoma and non-glaucoma was 65.9 (11.3) and 56.2 (10.3), respectively. Females constituted 52.7% of glaucoma cases and 59.2% of controls.

**TABLE 1:** Sample characteristics.

​	UK biobank (n = 324,713)			​	MAGGS (n = 4,519)		
​	Controls (322,545)	Cases (2,168)	*P* ^†^	​	Controls (4,274)	Cases (275)	*P* ^†^
Age (years)	57.1 (8.0)	63.0 (5.7)	<1E-300	Age (years)	56.2 (10.3)	65.9 (11.3)	1.81E-34
Female (%)	54.3%	48.4%	5.01E-08	Female (%)	59.2%	52.7%	0.04
PRS-POAG	−0.01 (1.00)	0.75 (0.99)	3.66E-218	PRS-POAG	−0.03 (0.99)	0.47 (0.97)	2.67E-15
PRS-IOP	−0.00 (1.00)	0.72 (1.00)	4.91E-203	PRS-IOP	−0.02 (1.00)	0.28 (1.03)	3.65E-06
PRS-VCDR	−0.00 (1.00)	0.47 (1.04)	9.91E-90	PRS-VCDR	−0.03 (0.99)	0.54 (0.97)	5.82E-19
PRS-RNFL	0.00 (1.00)	−0.08 (0.97)	9.54E-05	PRS-RNFL	0.01 (1.00)	−0.11 (1.02)	0.056
Education (%)	​	​	​	Education (%)	​	​	​
College or University degree	31.4%	28.8%	7.40E-09	≥12 years	34.5%	27.0%	0.01
Non-college qualification	50.4%	47.9%	​	7–11 years	22.0%	20.4%	​
None of the above	18.2%	23.3%	​	≤6 years	43.6%	52.6%	​
Missing	1.3%	1.8%	​	Missing	0.3%	0.4%	​
Household income (%)	​	​	​	Income (%)	​	​	​
< £18,000	22.5%	32.0%	2.01E-39	< $20,000	49.7%	59.8%	0.003
£18,000 to £30,999	25.5%	31.6%	​	$20,000 - $40,000	35.8%	31.8%	​
£31,000 to £51,999	26.4%	20.5%	​	> $40,000	14.5%	8.4%	​
£52,000 to £100,000	20.4%	13.5%	​	Missing	12.9%	13.1%	​
> £100,000	5.2%	2.4%	​	​	​	​	​
Missing	14.5%	18.0%	​	​	​	​	​

For continuous variables, mean and standard deviation for each group were shown. All PRSs, were standardized to have mean 0 and variance 1.

^†^ Chi-square test for categorical variables, Student’s t-test for continuous variables.

Abbreviation: IOP, intraocular pressure; MAGGS, mexican american glaucoma genetic study; PRS, polygenic risk score; POAG, primary open-angle glaucoma; RNFL, retinal nerve fiber layer thickness; VCDR, vertical cup-to-disc ratio.

Table 2 presents the single PRS association results (logistic regression analyses), adjusted for age and sex, among UKB and MAGGS participants. In the UKB cohort (Table 2a), the traditional clumping and thresholding (C + T) PRS for POAG (PRS-POAG-C + T) yielded an odds ratio (OR) of 1.71 (95% confidence interval [CI]: 1.64–1.79; *P* = 8.71 × 10^−141^). The SBayesRC-derived PRS for POAG (PRS-POAG, which integrates functional annotation information) showed a stronger association with an OR of 2.14 (95% CI: 2.05–2.24; *P* = 8.54 × 10^−269^). The SBayesRC-derived PRSs for IOP (PRS-IOP), VCDR (PRS-VCDR), and RNFL thickness (PRS-RNFL) also demonstrated significant associations with POAG. Similarly, in the MAGGS cohort (Table 3b), the PRS-POAG-MTAG had an OR of 1.44 (95% CI: 1.24–1.60; *P* = 1.17 × 10^−7^). PRS-VCDR showed the highest OR of 1.90 (95% CI: 1.66–2.17; *P* = 1.39 × 10^−20^). The PRS-POAG-C + T, PRS-IOP, PRS-POAG, and PRS-RNFL in MAGGS participants also exhibited significant associations with POAG.

**TABLE 2:** Single-PRS Logistic Regression Results. (a) UK Biobank participants.

​	OR (95% CI)	*P* Value
PRS-POAG-C+T	1.71 (1.64, 1.79)	8.71 × 10^−141^
PRS-POAG	2.14 (2.05, 2.24)	8.54 × 10^−269^
PRS-IOP	2.08 (2.00, 2.17)	1.04 × 10^−248^
PRS-VCDR	1.61 (1.54, 1.68)	9.50 × 10^−107^
PRS-RNFL	0.92 (0.89, 0.96)	2.52 × 10^−4^

**TABLE 3:** (b) MAGGS^†^ participants.

UK Biobank | MAGGS†	OR (95% CI)	*P* Value
PRS-POAG-C+T	1.64 (1.44, 1.88)	1.68 × 10^−13^
PRS-POAG-MTAG	1.44 (1.24, 1.60)	1.17 × 10^−7^
PRS-POAG	1.82 (1.59, 2.08)	2.79 × 10^−18^
PRS-IOP	1.45 (1.27, 1.65)	1.66 × 10^−8^
PRS-VCDR	1.90 (1.66, 2.17)	1.39 × 10^−20^
PRS-RNFL	0.85 (0.74, 0.96)	0.011

Results of single-PRS, logistic regression analyses, adjusted for age and sex.

† Unrelated MAGGS, participants (n = 4,108) were used. PRS-POAG, PRS-IOP, PRS-VCDR, and PRS-RNFL, were SBayesRC-derived. To prevent potential data leakage, MTAG-derived PRS (based on Craig et al.’s summary statistics, which used UK, Biobank data) was not applied to UK, Biobank participants. PRS-POAG-MTAG, was exclusively applied to MAGGS, study participants.

Abbreviations: C + T, clumping and thresholding; IOP, intraocular pressure; MAGGS, mexican american glaucoma genetic study; OR, odds ratio; PRS, polygenic risk score; POAG, primary open-angle glaucoma; RNFL, retinal nerve fiber layer thickness; VCDR, vertical cup-to-disc ratio.

Table 4 displays the results of the multiple PRS logistic regression analyses, assessing the combined association of PRSs with POAG and their relative importance in each cohort. In the UKB participants, PRS-POAG had an OR of 1.59 (*P* = 1.23 × 10^−74^), PRS-IOP showed the strongest association with an OR of 1.63 (*P* = 5.37 × 10^−89^), PRS-VCDR had an OR of 1.28 (*P* = 1.99 × 10^−27^), and PRS-RNFL exhibited an OR of 0.94 (*P* = 0.002), indicating a slight inverse association (larger PRS-RNFL indicated thicker RNFL and hence is protective). The OR for a simultaneous one-SD-unit-increase in all four standardized PRS variables was 3.11 (95% CI: 2.89–3.34). In the MAGGS cohort, PRS-POAG had an OR of 1.40 (*P* = 7.63 × 10^−5^), PRS-IOP an OR of 1.15 (*P* = 0.07), PRS-VCDR showed the strongest association with an OR of 1.64 (*P* = 2.04 × 10^−11^), and PRS-RNFL had an OR of 0.85 (*P* = 0.016), also suggesting an inverse relationship. The OR for a simultaneous one-SD-unit-increase in all four standardized PRS variables was 2.25 (95% CI: 1.79–2.82). These results highlight ancestry-specific differences in genetic contributions to POAG risk, with PRS-IOP contributing most significantly in the UKB dataset (European ancestry), whereas PRS-VCDR had the strongest contribution in the MAGGS dataset (Latino ancestry). We further evaluated potential collinearity among these PRSs using VIF (Supplementary Table 1) and XGBoost with SHAP-based feature importance (Supplementary Figure 2). Both methods indicated no collinearity issues, underscoring the robustness of our logistic regression analyses.

**TABLE 4:** Multiple logistic regression results.

​	UK biobank		MAGGS^†^	
​	OR (95% CI)	*P* Value	OR (95% CI)	*P* Value
PRS-POAG	1.59 (1.51, 1.67)	1.23 × 10^−74^	1.40 (1.18, 1.65)	7.63 × 10^−5^
PRS-IOP	1.63 (1.55, 1.71)	5.37 × 10^−89^	1.15 (0.99, 1.35)	0.07
PRS-VCDR	1.28 (1.23, 1.34)	1.99 × 10^−27^	1.64 (1.42, 1.90)	2.04 × 10^−11^
PRS-RNFL	0.94 (0.90, 0.98)	0.002	0.85 (0.74, 0.97)	0.016

Results of multiple logistic regression analyses, adjusted for age and sex, assessing the combined association of polygenic risk scores (PRSs) with primary open-angle glaucoma.

† Unrelated MAGGS, participants (n = 4,108) were used. PRS-POAG, PRS-IOP, PRS-VCDR, and PRS-RNFL, were SBayesRC-derived.

Abbreviations: IOP, intraocular pressure; MAGGS, mexican american glaucoma genetic study; OR, odds ratio; PRS, polygenic risk score; POAG, primary open-angle glaucoma; RNFL, retinal nerve fiber layer thickness; VCDR, vertical cup-to-disc ratio.

**FIGURE 1:**
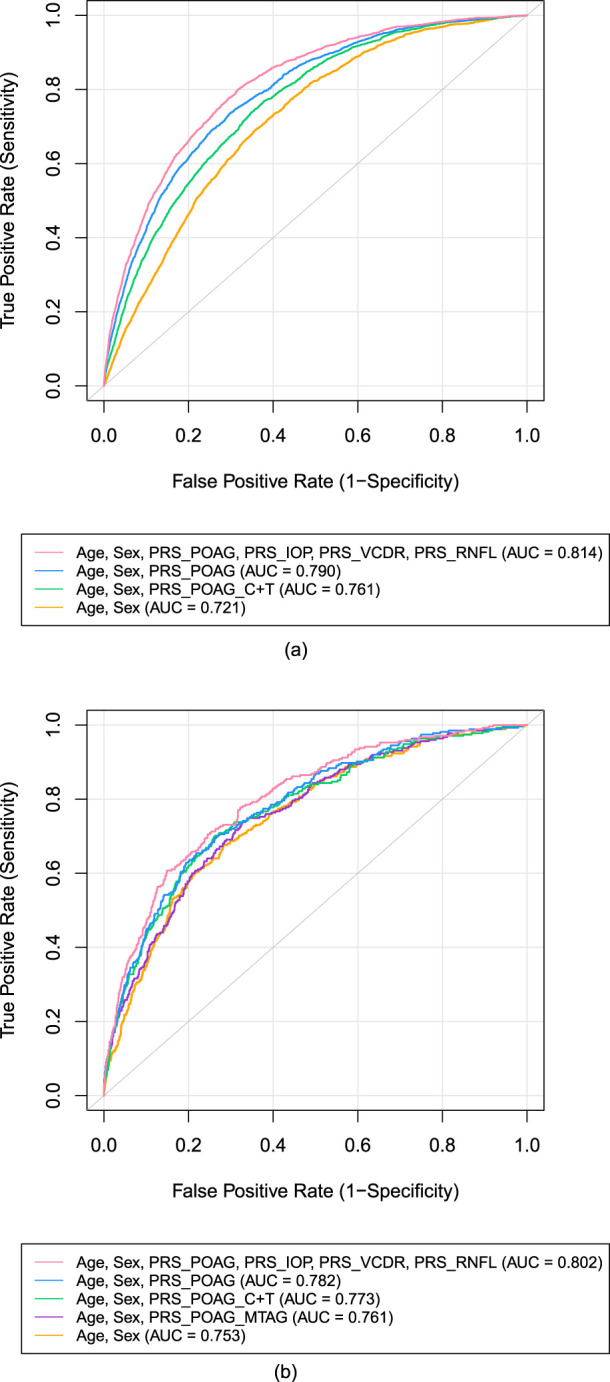
Receiver operating characteristic curves predicting POAG using polygenic risk scores. **(a)** UK Biobank participants. **(b)** MAGGS participants

We then examined the discriminatory ability of the PRSs (PRS-POAG, PRS-IOP, PRS-VCDR, and PRS-RNFL) for predicting POAG using penalized (L2) logistic regression with stratified 10-fold cross-validation and calculated the area under the receiver operating characteristic curve (AUC). Figure 1 illustrates these results. In UKB participants (Figure 1a), the baseline model including only age and sex had an AUC of 0.721 (95% CI: 0.711–0.730). Adding PRS-POAG-C + T increased the AUC to 0.761 (95% CI: 0.752–0.771), while incorporating the SBayesRC-derived PRS-POAG improved it to 0.790 (95% CI: 0.781–0.799), a 3% increase over the C + T approach (*P* = 1.13 × 10^−29^). Incorporating all four PRSs (PRS-POAG, PRS-IOP, PRS-VCDR, and PRS-RNFL) with adjustment for age and sex resulted in an AUC of 0.814 (95% CI: 0.806–0.823), representing a 9.3% improvement over the baseline model (*P* = 1.15 × 10^−112^) and a 2.1% improvement over the SBayesRC-derived PRS-POAG (*P* = 6.25 × 10^−28^). In MAGGS participants (Figure 1b), the baseline AUC was 0.753 (95% CI: 0.724–0.782), which increased to 0.761 (95% CI: 0.732–0.789) with PRS-POAG-MTAG, to 0.773 (95% CI: 0.744–0.802) with PRS-POAG-C + T, and to 0.782 (95% CI: 0.754–0.810) with PRS-POAG. The full model with all four PRSs (PRS-POAG, PRS-IOP, PRS-VCDR, and PRS-RNFL) achieved an AUC of 0.802 (95% CI: 0.775–0.828), providing a 5% enhancement over the baseline model (*P* = 5.25 × 10^−6^) and a 2% enhancement over the SBayesRC-derived PRS-POAG (*P* = 9.92 × 10^−4^). These findings indicate that incorporating multiple glaucoma-related PRSs significantly improves the prediction of POAG in both cohorts.

**FIGURE 2:**
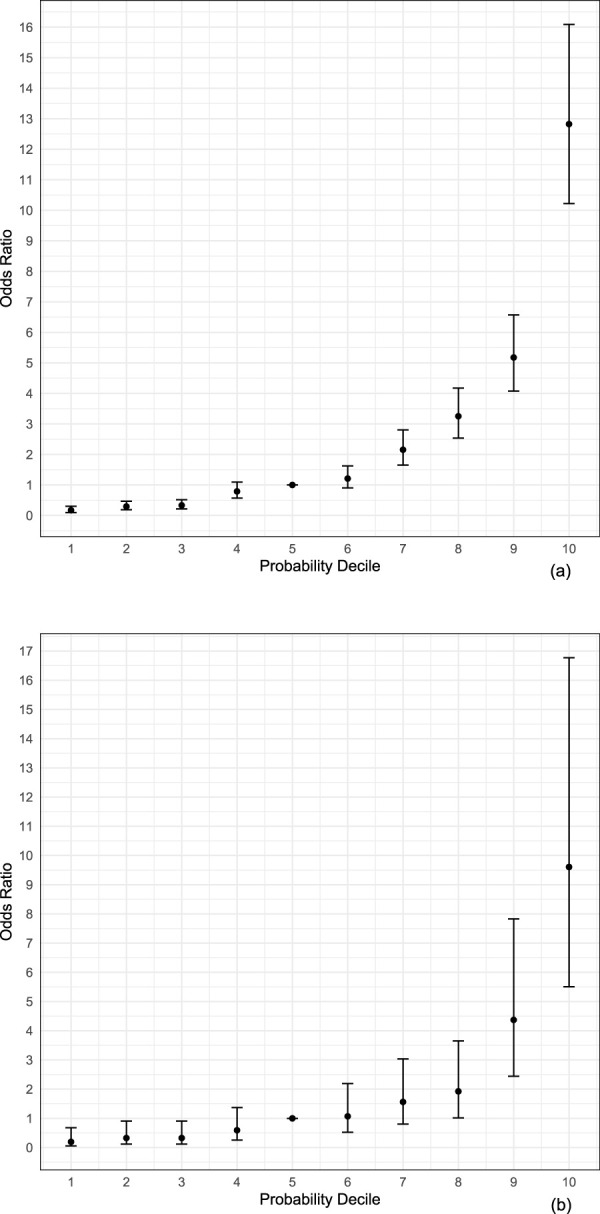
Association between polygenic probability risk score deciles and POAG risk. **(a)** UK Biobank participants. **(b)** MAGGS participants

We further evaluated POAG risk stratification using polygenic probability risk score (PPRS), integrating the effect of multiple PRSs of POAG, IOP, VCDR, and RNFL (with adjustment for age and sex), from our penalized 10-fold cross-validation logistic regression models. Analysis of PPRS revealed strong risk stratification capabilities (Figure 2). In the UKB cohort (Figure 2a), a clear trend emerged with increasing ORs across higher PPRS deciles. Individuals in the highest decile had 74.35-fold increased odds of POAG (95% CI: 43.87–126.02; *P* = 1.28 × 10^−57^) compared with those in the lowest decile, and 12.82-fold increased odds (95% CI: 10.22–16.09; *P* = 1.17 × 10^−107^) compared with those in the 5th decile. Similarly, in the MAGGS cohort (Figure 2b), participants in the highest decile demonstrated 49.34-fold increased odds of POAG (95% CI: 15.54–156.62; *P* = 3.69 × 10^−11^) compared with the lowest decile and 9.61-fold increased odds (95% CI: 5.50–16.77; *P* = 1.72 × 10^−15^) compared with the 5th decile. Most POAG cases (46.54% in UKB and 40.73% in MAGGS) fell within the highest decile category of PPRS (Supplementary Table 2). Taken together, these findings underscore the strong relationship between PPRS extreme deciles and POAG risk across UKB and MAGGS, highlighting the potential utility of the PPRS for POAG risk stratification.

## Discussion

Our study demonstrates that integrating multiple glaucoma-related traits with functional annotations substantially improves the polygenic prediction for POAG across diverse ancestries. Three key findings emerge from our analysis: First, our multi-modal PPRS approach achieved higher predictive accuracy, with AUC values of 0.814 (95% CI: 0.806–0.823) in Europeans and 0.802 (95% CI: 0.775–0.828) in Latinos, compared to our POAG single-trait models. This improvement likely reflects the complex genetic architecture of POAG, where multiple biological pathways contribute to disease development. Age and sex accounted for a substantial proportion of predictive performance (AUC = 0.721 in Europeans and 0.753 in Latinos), with PRS contributing additional discriminatory ability. Second, we identified ancestry-specific differences in the relative contributions of component PRSs. In our European samples, IOP showed the strongest association, whereas VCDR was most strongly associated in our Latino samples. This finding underscores the importance of population-specific risk assessment and suggests distinct genetic mechanisms underlying POAG across ancestral groups. Third, the strong risk stratification achieved by our PPRS, demonstrating over 74.4-fold and 49.3-fold risk differences between extreme deciles in Europeans and Latinos, respectively, suggests significant clinical utility. Importantly, unlike approaches that collapse multiple traits into a single combined score (e.g., via multi-trait analysis of GWAS), our framework preserves trait-specific PRS contributions, enabling the identification of ancestry-specific differences in the genetic architecture underlying POAG risk.

One of the most direct potential clinical applications of PRS is identifying high-risk individuals. MacGregor et al. (2018), using an IOP PRS, reported an increased glaucoma risk (OR = 5.6, 95% CI: 4.1–7.6) comparing the top and bottom deciles. Craig et al. (2020), employing an MTAG approach for their POAG PRS, observed a higher OR of 14.9 (95% CI: 10.7–20.9) for comparing the top and bottom deciles. Gao et al. (2019) found an OR of 6.3 for their top IOP PRS quintile *versus* the bottom quintile comparison, with approximately 40.6% of POAG cases found in the top quintile. Similarly, de Vries et al. (2025) identified about 50% of cases in their top glaucoma PRS quintile. Our study substantially improves upon these results, demonstrating ORs of 74.4 (95% CI: 43.9–126.0) in Europeans and 49.3 (95% CI: 15.5–156.6) in Latinos when comparing extreme deciles of our PPRS. Additionally, we identified 65.7% and 62.2% of glaucoma cases within the top quintile of our PPRS scores for Europeans and Latinos (Supplementary Table 2), respectively, representing a substantial improvement over previous studies.

While the improvement in AUC from the best single PRS (PRS-POAG, AUC = 0.790) to the full PPRS model (AUC = 0.814) may appear modest in absolute terms (ΔAUC = 0.024), such increments are consistent with prior reports in complex disease genetics and can nonetheless have important implications in practice. In our study, the PPRS not only improved overall discrimination but also strengthened risk separation at the extremes of the distribution, with substantially elevated odds of glaucoma observed in the top decile compared with the bottom decile. Most importantly, the full PPRS model identified 12.1% more cases in the top decile than the PRS-POAG model. Although the absolute improvement in AUC is modest, even small gains in discrimination can meaningfully enhance risk stratification, particularly in identifying high-risk individuals who may benefit from targeted screening or early intervention. We therefore view the statistically significant increment in AUC as both supportive of the additive value of integrating multiple glaucoma-related PRSs and a foundation for further improvement when combined with imaging, electronic health record, or other multimodal data in future predictive models.

The observation that PRS-IOP contributed most strongly to prediction in Europeans, whereas PRS-VCDR appeared more influential in Latinos, is intriguing but should be interpreted with caution. These differences may partly reflect disparities in sample size and statistical power across discovery GWAS. Cohort-specific factors such as recruitment differences, phenotype definitions, and environmental exposures may also have influenced the relative performance of the PRSs. In the UKB, POAG was identified through ICD-10 codes recorded during routine clinical care, whereas in MAGGS, all participants received standardized ophthalmic examinations by glaucoma specialists. This difference likely contributes to the markedly higher POAG prevalence in MAGGS (6.0%) compared with the UKB (0.7%), as undiagnosed cases in the UKB may be misclassified as controls. Such differential misclassification could attenuate PRS–POAG associations in the UKB, and may also influence which trait-specific PRSs appear most predictive in each cohort. In particular, the observation that IOP-related genetic variation contributes most strongly in Europeans whereas VCDR-related variation dominates in Latinos could partly reflect detection bias: POAG cases identified through routine care (UKB) may be enriched for IOP-driven disease, while comprehensive screening (MAGGS) captures a broader spectrum of disease mechanisms including structural optic nerve changes. Nevertheless, prior studies have shown that IOP PRS significantly improves discrimination for glaucoma in Europeans (Gao et al., 2019), and over 72% of known POAG risk loci are also associated with IOP in predominantly European datasets (Gharahkhani et al., 2021), underscoring the key role of IOP in POAG pathogenesis in this ancestry group. At the same time, epidemiological studies report variation in POAG presentation across populations (Varma et al., 2004b): in Latinos, the majority of cases occur with IOP <21 mmHg, with elevated IOP (>21 mmHg) observed in only ∼18% of cases. This pattern is consistent with stronger predictive value of VCDR-based genetic risk in the MAGGS cohort. The borderline non-significance of PRS-IOP in the MAGGS multiple regression model (*P* = 0.07) underscores the need for caution. The relatively modest sample size of the MAGGS cohort may limit statistical power for precise estimate. Therefore, observed differences in PRS contributions should be interpreted in the context of these limitations and require validation in larger and more diverse populations.

Our study has several strengths, notably the use of a comprehensive multi-trait PPRS approach that integrates multiple glaucoma-related traits and leverages functional annotations to improve prediction accuracy. By evaluating PRS performance in both European and Latino populations, we address the crucial need for inclusive and representative genetic research, especially among traditionally understudied groups (Martschenko et al., 2023). However, our study also has limitations. Due to resource constraints, we included only European and Latino populations in this study. Expanding future research to additional ancestry populations would enhance generalizability and ensure broader applicability of our findings. In addition, the smaller sample size of the MAGGS Latino cohort limits the precision of effect estimates within this ancestry, underscoring the need for replication in larger datasets. Differences in healthcare access and screening intensity between cohorts may result in differential case ascertainment, potentially leading to under diagnosis in population-based datasets such as UKB. This may attenuate observed associations and predictive performance. Because the study population is predominantly older, the applicability of these findings to younger individuals remains uncertain. Future studies in younger cohorts are needed to evaluate early-life predictive utility. Furthermore, our PRS construction was limited to common variants. Incorporating rare variants, similar to previous reports on rare variants associated with IOP (Gao et al., 2022), warrants further investigation. Additionally, compared with recent large-scale efforts that leverage discovery samples exceeding 600,000 individuals (Han et al., 2023), our GWAS discovery samples are substantially smaller; however, the purpose of our study is to demonstrate the added value of a multi-trait integration framework rather than to maximize single-trait PRS performance through sample size alone. Future studies combining our multi-trait approach with larger multi-ancestry discovery datasets may yield further improvements. To demonstrate ancestry-related differences in PRS contribution, we used ancestry-stratified PRS models. But real-world clinical populations typically comprise individuals of mixed and diverse ancestries. To improve clinical applicability, future work should explore continuous ancestry measures. The current model assumes additive effects of trait-specific PRSs. However, non-linear relationships or interactions among traits (e.g., IOP and VCDR) may further improve prediction. Future studies using more flexible modeling frameworks may help capture these complex relationships.

It is noteworthy that the MTAG-derived PRS did not outperform the single-trait GWAS PRS in our analysis. Several factors likely contributed. First, we applied MTAG only to the MAGGS Latino samples, where the smaller cohort size may limit stable estimation. Second, we constructed PRS using the state-of-the-art SBayesRC framework, which leverages functional annotations to increase power. Finally, although the recently published large-scale POAG MTAG GWAS (Han et al., 2023) included >600,000 participants and could potentially offer improved power, only p-values (and not SNP effect sizes) were released, precluding its direct use for PRS derivation in this study. Notably, our multi-trait PPRS approach differs from MTAG by combining individual trait-specific PRSs at the prediction model level, which enables the assessment of differential trait contributions across ancestries. SNP-level multi-trait approaches such as MTAG improve statistical power by jointly modeling variant-level effects across traits. In contrast, our PRS-level integration combines trait-specific genetic predictors, enabling flexible incorporation of heterogeneous data sources and facilitating interpretation of trait contributions. These approaches are complementary, with PRS-level methods prioritizing interpretability and flexibility, and SNP-level methods maximizing statistical efficiency.

In conclusion, our multi-trait PPRS approach, combining multiple glaucoma-related traits and incorporating functional annotations, represents a significant advancement in polygenic risk prediction for POAG. By retaining trait-specific PRS contributions rather than collapsing them into a single score, our framework uniquely reveals that the genetic determinants of POAG risk differ across ancestries, with IOP-related genetic variation contributing most strongly in our European samples and VCDR-related variation in our Latino samples. The robust performance across different ancestral groups and substantial risk stratification capabilities suggest considerable potential for enhancing early detection and preventative strategies. By facilitating the identification of individuals at high genetic risk, our approach has significant implications for timely clinical intervention and could ultimately reduce the public health burden of POAG.

## Data Availability

The SNP weight data used in this study are available at https://github.com/xraygao/PPRS.
