# Correction: Enhancing glaucoma prediction across ancestries: integrating functional annotation into multi-trait polygenic risk scores

**DOI:** 10.3389/fgene.2026.1942473

**Published:** 2026-08-03

**Authors:** Xiaoyi Raymond Gao

**Affiliations:** 1 Department of Ophthalmology and Visual Sciences, The Ohio State University, Columbus, OH, United States; 2 Department of Biomedical Informatics, The Ohio State University, Columbus, OH, United States

**Keywords:** diverse ancestry, genetic prediction, glaucoma, polygenic risk score, risk stratification

In the **acknowledgements**, the UK Biobank application number was omitted. The following statement should have been included: “This research has been conducted using the UK Biobank Resource under Application Number 23424.”

The original version of this article has been updated.

