# Supplementary material for: Enhancing glaucoma prediction across ancestries: integrating functional annotation into multi-trait polygenic risk scores
Source: Front Genet. 2026 May 7;17:1842316. doi: 10.3389/fgene.2026.1842316 (PMC13190481; doi:10.3389/fgene.2026.1842316)
Supplement: Supplementary file 1 [file DataSheet1.docx]

**Supplementary Table 1. Variance Inflation factors for polygenic risk scores in our logistic regression models**

1. UK Biobank

| Age | Sex | PRS-POAG | PRS-IOP | PRS-VCDR | PRS-RNFL |
| --- | --- | --- | --- | --- | --- |
| 1.002 | 1.002 | 1.347 | 1.240 | 1.112 | 1.003 |

1. MAGGS^†^

| Age | Sex | PRS-POAG | PRS-IOP | PRS-VCDR | PRS-RNFL |
| --- | --- | --- | --- | --- | --- |
| 1.024 | 1.006 | 1.555 | 1.371 | 1.166 | 1.004 |

Displayed are the variance inflation factors for the polygenic risk scores (PRSs) used in our logistic regression analyses of a) UK Biobank; b) MAGGS. ^†^Unrelated MAGGS participants were used. PRS-POAG, PRS-IOP, PRS-VCDR, and PRS-RNFL were SBayesRC-derived. These variant inflation factor values are only slightly above 1 and all remain well below the typical cutoff of 5, indicating no collinearity issues.

Abbreviations: IOP, intraocular pressure; MAGGS, Mexican American Glaucoma Genetic Study; PRS, polygenic risk score; POAG, primary open-angle glaucoma; RNFL, retinal nerve fiber layer thickness; VCDR, vertical cup-to-disc ratio.

**Supplementary Table 2. Number of POAG Cases in Each Decile Category**

(a) UK Biobank

|  | Predicted Probability Risk Score Decile | | | | | | | | | |
| --- | --- | --- | --- | --- | --- | --- | --- | --- | --- | --- |
|  | 1 | 2 | 3 | 4 | 5 | 6 | 7 | 8 | 9 | 10 |
| POAG, n (%) | 14  (0.65) | 24  (1.11) | 27  (1.25) | 64  (2.95) | 81  (3.74) | 98  (4.52) | 174 (8.03) | 262  (12.08) | 415  (19.14) | 1009  (46.54) |

(b) MAGGS

|  | Predicted Probability Risk Score Decile | | | | | | | | | |
| --- | --- | --- | --- | --- | --- | --- | --- | --- | --- | --- |
|  | 1 | 2 | 3 | 4 | 5 | 6 | 7 | 8 | 9 | 10 |
| POAG, n (%) | 3  (1.09) | 5  (1.82) | 5  (1.82) | 9  (3.27) | 15  (5.45) | 16  (5.82) | 23  (8.36) | 28  (10.18) | 59  (21.45) | 112  (40.73) |


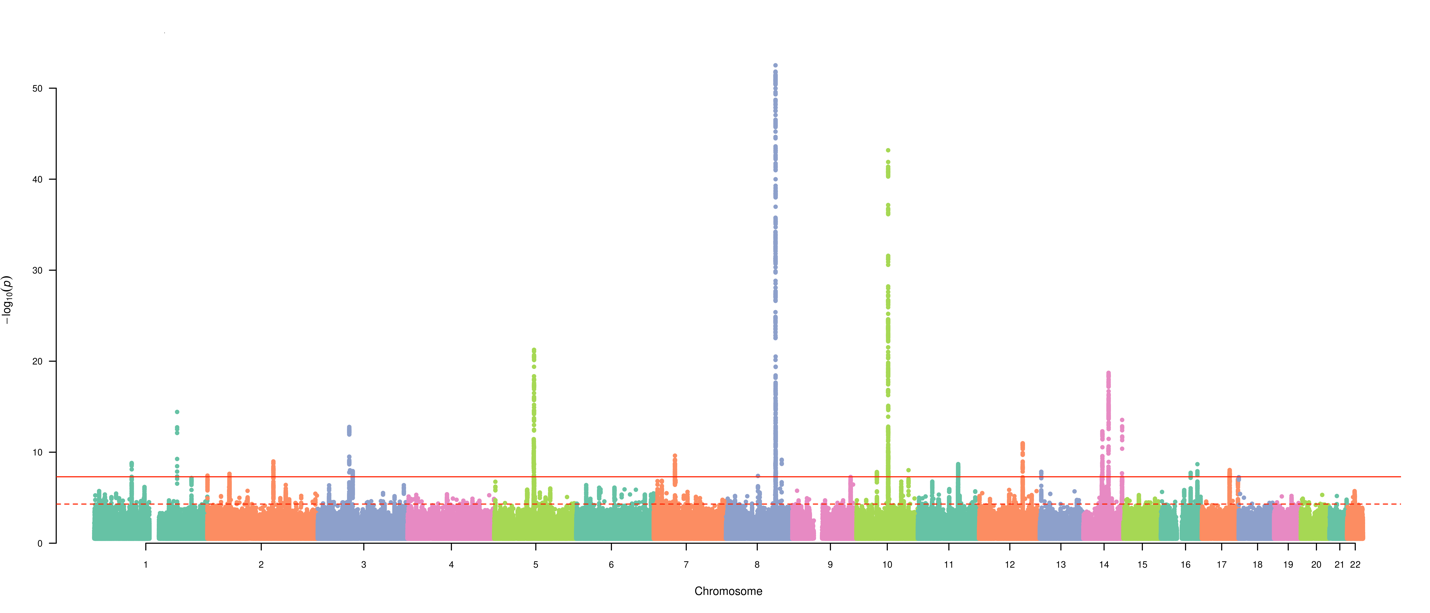


**Supplementary Figure 1. Manhattan Plot Displaying the –log10(*P* values) for the Association Between RNFL Thickness and Genome-wide Genetic Variants**

Solid and dotted horizontal lines represent genome-wide significant associations (*P* < 5 ×10^-8^) and suggestive associations (*P* < 5 ×10^-5^), respectively. Genetic variants are plotted by chromosomal position.

1. UK Biobank participants

1. MAGGS participants

**Supplementary Figure 2. SHAP Feature Importance for XGBoost Models**

Bar charts illustrating feature importance, as evaluated via SHapley Additive exPlanations (SHAP) values, for the (A) UK Biobank and (B) Mexican American Glaucoma Genetic Study (MAGGS) cohorts. The y-axis displays the ranked features, and the x-axis shows the average impact on the model’s output. All PRSs (PRS-POAG, PRS-IOP, PRS-VCDR, and PRS-RNFL) were SBayesRC-derived. In UKB participants (European ancestry), PRS-IOP and PRS-POAG both rank highly among the PRSs, whereas in MAGGS participants (Latino ancestry), PRS-VCDR shows the highest importance among the PRSs. These results align with the logistic regression findings, confirming ancestry-specific differences in genetic contributions to POAG risk.

Abbreviations: IOP, intraocular pressure; RNFL, retinal nerve fiber layer; VCDR, vertical cup-to-disc ratio.
